# Case Study: Analyzing CFTR Mutations and SNPs in Pulmonary Fibrosis Patients with Unclear Symptoms

**DOI:** 10.1155/2024/8836342

**Published:** 2024-09-23

**Authors:** Sahar Yousaf, Sumaira, Iqbal Bano, Atia Rehman, Samra Kousar, Muhammad Usman Ghani, Mariam Shahid

**Affiliations:** ^1^ Centre of Excellence in Molecular Biology University of the Punjab, Lahore, Pakistan; ^2^ Children Hospital University of Child Health Sciences, Lahore, Pakistan; ^3^ Center for Applied Molecular Biology University of the Punjab, Lahore, Pakistan

## Abstract

Cystic fibrosis (CF) is a genetic monogenic disorder inherited in an autosomal recessive manner, marked by persistent airway infections in the endobronchial region. This condition leads to the gradual development of bronchiectasis and, ultimately, respiratory failure, emerging as the primary cause of mortality in individuals diagnosed with CF. Diagnosis is done depending on the patient's symptoms and lung radiological findings like chest X-rays and CTs. In younger patients and children, diagnosis becomes difficult due to overlapping symptoms with other diseases such as CF which is a rare genetic disease in our population. Diagnosis of CF usually relies on characteristic symptoms, a family history of CF, and an abnormal sweat chloride test, but in children, low sweat production during testing leads to false negative results. In this case report, a suspected patient with ambiguous respiratory symptoms underwent a comprehensive investigation revealing elevated CRP levels, TLC, and characteristic pulmonary manifestations on chest X-ray, suggesting cystic fibrosis. Despite negative sweat chloride tests, the patient was analysed for potential candidate SNPs and was also tested for potential CFTR mutations to rule out CF, genetic analysis confirmed the diagnosis. Genetic testing plays a crucial role in diagnosing cystic fibrosis, even when traditional tests are inconclusive. Specific mutations like Δ508 deletion and rs213950 guide personalized treatment. Consanguinity and family history highlight genetic predisposition, while environmental factors may influence symptom onset. Further research is needed to understand these complexities and improve diagnostic and treatment approaches.

## 1. Introduction

Respiratory disease occurs in more than one billion people worldwide, and unidentified lung disease is one of the main causes of mortality and morbidity among many patients. Chronic lung disorders also occur in adults, but young children and infants are more affected [1].

Respiratory diseases are affected by age, smoking history, gender, and exposure to certain chemicals [2]. It is difficult to diagnose complex lung disease in children due to a lack of molecular testing. Cystic fibrosis and interstitial lung disease are major ones in these because of overlapping symptoms and lung fibrosis in both diseases. In some cases, when there is no family history and the molecular testing for CF also comes back negative, these patients may be suspected of having interstitial lung diseases and should be evaluated for potential candidate genes associated with these diseases.

**Figure 1:**
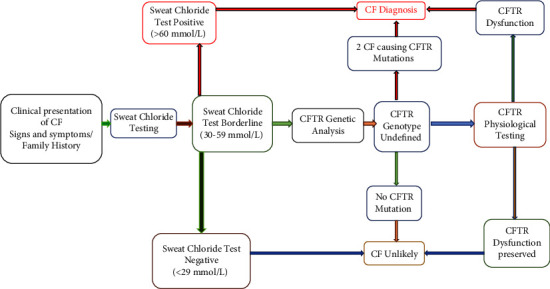
Simplified algorithm for how consensus statements should be applied to individuals suspected of having cystic fibrosis. From Reference [8], with permission. CF = cystic fibrosis; CFTR = cystic fibrosis transmembrane regulator [6].

Cystic fibrosis is a genetic monogenic disorder inherited in an autosomal recessive manner, marked by persistent airway infections in the endobronchial region. This condition leads to the gradual development of bronchiectasis and, ultimately, respiratory failure, emerging as the primary cause of mortality in individuals diagnosed with CF [3, 4]. CF is a prevalent and serious multisystem disorder with life-threatening implications [5]. Despite advancements in understanding the natural progression of CF, which have informed treatment strategies and contributed to enhanced pulmonary well-being and prolonged life expectancy for individuals with the disorder, respiratory complications continue to be the primary source of morbidity and mortality in CF patients [6]. It was initially documented in the global medical community in 1949 [7]. It is a potentially life-threatening hereditary condition resulting from mutations in the gene responsible for encoding the CF transmembrane conductance regulator (CFTR) protein. This protein serves as an anion channel expressed at the apical membrane of secretory epithelia [8]. CFTR is a crucial protein comprising 1480 amino acids, known for its role in regulating anion channels and maintaining electrolyte balance and fluid movement across mucosal surfaces, particularly in the lungs. Dysfunction of CFTR leads to a disruption in surface liquid hydration along the airways, making it challenging for the body to effectively clear microorganisms. Consequently, individuals with CFTR abnormalities experience chronic airway infections, marked by intense local inflammation and excessive mucus production. This cycle perpetuates a decline in lung function, often resulting in premature mortality, typically occurring in the third or fourth decade of life due to respiratory failure [9]. In adults, these findings are promising for diagnosis, but in younger patients and children, diagnosis becomes difficult in most cases. Many chronic lung diseases have overlapping symptoms which make them difficult to diagnose e.g., in cystic fibrosis which is a rare genetic disease in our population, no molecular testing is done as part of diagnosis, and sweat chloride test is done as a screening test for suspected patients. Diagnosis of CF usually relies on characteristic symptoms, a family history of CF, and an abnormal sweat chloride test, but in children, low sweat production during testing leads to false negative results. Another major group of respiratory disease is interstitial lung disease in which genetic and epigenetic factors are known to play an important role (Figure 1).

In our population, these cases also remain misdiagnosed due to a lack of molecular testing facilities. As different respiratory genetic diseases have similar symptoms and remain undiagnosed in our population, there is a need for specific molecular testing for timely diagnosis of these diseases. Genetic testing will not only help in disease diagnosis and proper treatment of patients but also plays a role in carrier identification, identification of new variants, and genetic counselling of patients.

Both genetic and environmental factors play essential role in disease development and prognosis [10]. These genetic and epigenetic factors are significant in developing fibrotic process, but contribution of the variants and their interaction with the putative external factors has yet to be clarified [11].

The emergence and development of clinical signs of lung disease in CF can vary significantly. While respiratory issues are rare in newborns, older infants may exhibit persistent coughing, recurrent wheezing, rapid breathing, and frequent lung infections. In regions lacking neonatal CF screening, these respiratory symptoms are often mistaken for asthma or recurring bronchitis, leading to delays in appropriate treatment. As CF-related lung disease advances, patients may also experience breathlessness and reduced exercise tolerance.

## 2. Case Presentation

The patient, a 7-year-old female, SLD-10, was admitted to Children's Hospital Lahore presenting with a complex medical history. She has complained of persistent cough, fever, and vomiting. Notably, her family history included her grandfather's demise due to asthma, suggesting a genetic predisposition to respiratory issues. Patient's symptoms, initially vague, prompted a comprehensive investigation to unravel the underlying cause.

**Figure 2:**
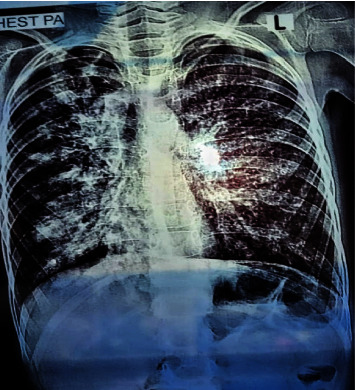
X-ray imaging of the patient reveals characteristic pulmonary manifestations, including hyperinflation, bronchiectasis, and mucus plugging, reflect the disease's impact on airway structure and function.

Medical examinations revealed elevated C-reactive protein (CRP) levels at 14.23 mg/dL (Normal: less than 0.3 mg/dL), indicative of systemic inflammation. Additionally, her Total Leukocyte Count (TLC) was raised 14.2 × 10^3^/*µ*L (Normal: 4.0–11.0 × 10^3^/*µ*L), pointing towards an active inflammatory response within her body. Sputum culture and Gram stain analysis revealed significant findings (Table 1). Sputum culture displayed heavy growth of *Staphylococcus aureus*, a common pathogen associated with respiratory infections, along with a few colonies of *Pseudomonas aeruginosa*, indicating a mixed bacterial infection. Gram stain examination further corroborated these findings by showing numerous pus cells, indicative of an inflammatory response to the bacterial invasion. These microbiological findings suggest a complex pulmonary infection, necessitating tailored antibiotic therapy targeting both pathogens to effectively manage the patient's respiratory condition and prevent further complications. The chest X-ray further unveiled a distinct pattern consistent with fibrosis. Radiographic findings included bronchiectasis, peribronchial thickening, increased lung markings, hyperinflation of lung fields, and evident mucous plugging (Figure 2).

HRCT findings suggested complete collapse of right lung with extensive bronchiectasis, volume loss mediastinal shift and hyperinflation of the left lung with herniation across in right hemothorax favors opportunistic infection in the right lung (Supplementary Figure S1). Despite exhibiting suspicious symptoms indicative of cystic fibrosis, the patient's sweat chloride test has yielded negative results in three consecutive evaluations. Remarkably, in the presence of a sweat chloride test yielding 0.00 mEq/L by the conductivity method (Table 1), the genetic analysis revealed mutations consistent with cystic fibrosis, highlighting the complexity and variability of this heterogeneous disorder. This underscores the critical role of genetic testing in diagnosing cystic fibrosis, particularly in cases where traditional diagnostic measures may yield inconclusive or contradictory results. Further clinical management will necessitate tailored therapeutic interventions guided by the identified genetic mutations to optimize patient care and outcomes.

Genetic studies played a pivotal role in confirming the diagnosis. CFTR mutations were identified, with the presence of delta 508, a three base pair deletion, and a Single Nucleotide Variant (SNV) at position 213950. These mutations not only substantiated the diagnosis but also provided valuable insights into the genetic basis of the patient's condition, connecting it to her family's history of lung disorders. Early intervention, coupled with a holistic, multidisciplinary approach, was deemed essential for optimizing her long-term outcomes and ensuring an improved quality of life. The suspected patient was analysed for potential association with candidate SNPs. Three base pair deletions (Delta 508) were seen at positions 117559591 to 117559593 in the patient ID (SLD10). This patient also shows a risk allele for rs213950. Pathogenicity of rs213950 was predicted by using ENTERPRISE X and I-mutant bioinformatics tools which showed that this variant has a deleterious effect (ENTERPRISE-X = 0.93948, I-mutant = decrease protein stability). This patient had a positive family history of respiratory diseases with the consanguineous marriage of parents. This mutation is present at the exon 10 of the cystic fibrosis transmembrane regulator gene (CFTR) and deletion of three base pairs causes loss of phenyl alanine amino acid at position 508. Thus, it is termed as delta F508. As specific treatment lines have been identified for this type of mutation, thus molecular testing should also be done as part of diagnosis along with another test, so effective treatment can be given to patients on time. This in-depth understanding of such a case not only facilitated a precise diagnosis but also laid the foundation for a comprehensive and personalized treatment strategy tailored to her unique medical profile.

## 3. Methods

A written consent form was signed by the guardian of patient. History of the patient was taken at Children Hospital and Institute of Child Health, Lahore. For the study purpose, an open-ended questionnaire was planned. It consists of personal information such as age, name, gender, caste, weight, and residence area. Risk factors such exposure to dust, smoke, and other allergens were also noted. Family history and disease-related parameters including any previous genetic analysis results, CT, or HRCT findings results were taken. Primers were designed by Primer3 software and optimized using different strategies including different annealing temperatures and reagents' concentrations. Optimization was done by using gradient PCR. Peripheral blood sample was taken, and genomic DNA was extracted by using the phenol chloroform isoamyl alcohol (PCI) method. Extracted genomic DNA was quantified with the help of agarose gel electrophoresis. PCR (Polymerase Chain Reaction) was used for the amplification of required genomic regions in disease-associated genes, from 25 ng of DNA. The amplified products were purified by using the exo-nuclease 1 and shrimp alkaline phosphate, EXO-SAP purification method. Purified products were amplified with sequencing PCR protocol using the ABI 3130xl analyser and Sanger's sequencing principle based on chain termination. DNA sequences obtained from capillary electrophoresis were converted into BioEdit format to make them align and use them for further manipulations. Geneious prime software was used for genomic and sequence analysis.

## 4. Results

**Figure 3:**
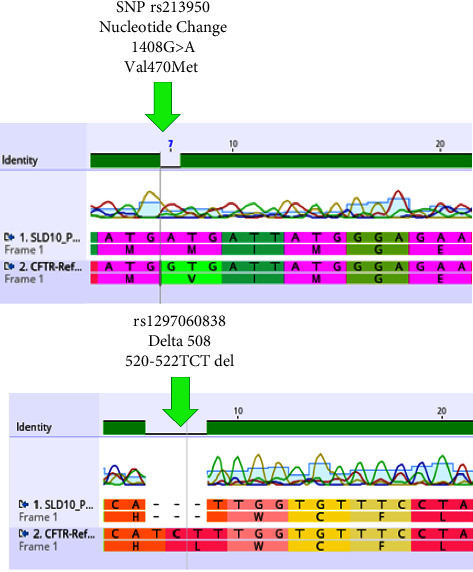
Electropherogram revealing risk allele variant for rs213950 nucleotide change at 1408 G > A and valine substituted with methionine at position 470, Δ508 deletion. Three base pair deletion seen at position 520–522 TCT deletion corresponding to CFTR mutation. (G = Guanine, A = Adenine, T = thymine, C = Cytosine).

The suspected patient of cystic fibrosis was analysed for potential association with candidate SNPs. Three base pair deletions (Delta 508) were seen at position 520–522 TCT deletion in the patient. This patient also shows a risk allele for rs213950 nucleotide change 1408 G > A and valine substituted with methionine at position 470 (Figure 3). This Δ508 deletion and risk allele confirmed that the patient is suspected for cystic fibrosis. The predominant mutation found in the gene CFTR linked to CF leads to the removal of three base pair deletions (TCT) in the CFTR protein. This alteration hinders the proper transportation of the CFTR protein to the cell's plasma membrane.

## 5. Discussion and Conclusion

In this study, suspected patient of cystic fibrosis was analysed for potential association with candidate SNPs. The prevalence of the disease in the Pakistani population, which comprises individuals of diverse ethnic backgrounds, is unknown however it is expected to be high considering the high cousin marriage rate. It is considered an underdiagnosed disease in Pakistan owing to the lack of diagnostics for confirmation of CF in this country. Most of the patients reported are diagnosed based on suggestive clinical features. Since phenotypic expression of the disease is related to genotype, elucidation of these mutations would be critical for its understanding and management in this population [12].

Three base pair deletion (Delta-508) was seen at position 520–522 TCT-deletion in the patient. This patient also shows a risk allele for rs213950 nucleotide change 1408 G > A and Valine substituted with Methionine at position 470. This Δ508 deletion and risk allele confirmed patient is suspected for Cystic fibrosis. This patient had positive family history of respiratory diseases with consanguineous marriage of parents similar to cases reported in a study by Al Oraimi et al. [13]. This mutation has already been reported in Pakistani population in a study at Aga khan university [14, 15]. A study on Jordanian population also showed ΔF508 as most frequent mutation in cystic fibrosis patients [16]. As specific treatment line have been identified for this type of mutation, thus molecular testing should also be done as part of diagnosis along with other test so that effective treatment can be given to the patients on time [17, 18].

The patient, carrying both the Δ508 deletion mutation and SNP rs213950, exhibited symptoms from birth and was from a rural area. The late onset of symptoms in these patients can be due to some other environmental exposure that triggers the symptoms in genetically predisposed persons. Consanguineous marriage and family history of respiratory disease play a specific role, in the relatively high incidence of CF as reported in a study in Oman where consanguinity was observed in about 70% of cases [13]. Variation with rs213950 with cytogenetic location 7q31.2 has already been reported in a study on Chinese and Caucasians in relation to CF patients by Ni et al. [19, 20]. Protein change of Val470Met was present at this site. Its clinical significance is considered to be benign or likely benign in Cystic Fibrosis and CFTR related disease according to previous studies. In our study, this variation was observed in patients having symptoms of cough, fever and respiratory difficulty suspected of fibrosis which also suggest this variation to be associated with CFTR related disease. Thus, further studies should be done with large number of samples so we can conclude this variation as pathogenic in our population.

## Figures and Tables

**Table 1:** Summary of medical examinations.

Parameters	Value
CRP	14.23 mg/dL
TLC	14.2 × 10^3^/*µ*L
Differential count	
Neutrophils	71.4%
Lymphocyte	25%
Monocyte	3.3%
Eosinophils	0.0%
Sweat chloride test	0.00 mEq/L
Pulmonary function tests (FEV1/FVC)	0.65
Height	2.5 feet
Weight	13 kg
Age	6 years
BMI	22.38

## Data Availability

Access to data is permitted with the authors' permission.

## Abbreviations

CF: Cystic fibrosis
CFTR: Cystic fibrosis transmembrane regulator gene
TLC: Total leukocyte count
CRP: C-reactive protein
SNP: Single nucleotide polymorphism
DNA: Deoxyribonucleic acid
CT: Computed tomography scan
PCI: Chloroform isoamyl alcohol
PCR: Polymerase chain reaction
HRCT: High-resolution computed tomography
SNV: Single nucleotide variant
EXO-SAP: Exo-nuclease 1 and shrimp alkaline phosphate
NBS: Newborn screening
MVCC: Mutation of varying clinical consequence
NPD: Nasal potential difference
ICM: Intestinal current measurement.

## Supplementary Materials

Supplementary Materials Supplementary Figure S1: HRCT findings indicating a complete collapse of the right lung, accompanied by extensive bronchiectasis and volume loss. There is also a mediastinal shift, along with hyperinflation of the left lung, which is herniating into the right hemothorax. These findings suggest the presence of an opportunistic infection in the right lung.
