# Supplementary material for: Case Study: Analyzing CFTR Mutations and SNPs in Pulmonary Fibrosis Patients with Unclear Symptoms
Source: Case Rep Med. 2024 Sep 23;2024:8836342. doi: 10.1155/2024/8836342 (PMC11442034; doi:10.1155/2024/8836342)
Supplement: Supplementary Materials — Supplementary Figure S1: HRCT findings indicating a complete collapse of the right lung, accompanied by extensive bronchiectasis and volume loss. There is also a mediastinal shift, along with hyperinflation of the left lung, which is herniating into the right hemothorax. These findings suggest the presence of an opportunistic infection in the right lung. [file 8836342.f1.pdf]

THE CHILDREN'S HOSPITAL & THE UNIVERSITY OF CHILD HEALTH SCIENCE
FEROZPUR ROAD, LAHORE (PAKISTAN)
Tel (042) 9230901-23, Fax (042) 9230358
DIAGNOSTIC RADIOLOGY DEPARTMENT

MR No: 5602		Scan Date: 11.06.24	
CT Ref. No. 13549		Reporting Date: 12-Jun-24	
PATIENT NAME:	[redacted]	AGE/SEX:	7 Years/ F
AREA SCANNED:	HRCT Chest	REF BY:	

CT REPORT

Clinical Features: - K/C of cystic fibrosis.

Discussion: High resolution thin slice axial images are acquired through the chest and parenchymal windows.

There is complete collapse of right lung with extensive bronchiectatic changes. Ipsilateral mediastinal shift towards right. Elevation of right hemi diaphragm suggestive of volume loss.

Hyperinflation of left upper and lower lobe crossing midline with shift of anterior junction line towards right.

Multiple groundglass and soft tissue density pulmonary nodules demonstrating tree in Bud in left lung with subsegmental area of groundglass density in left lung with bronchiectatic changes in left perihilar l location.

No evidence of mosaic attenuation.

No interstitial/reticular thickening noted. No evidence of abnormal parenchymal cyst or subpleural honeycombing. Trachea, mainstem bronchi and their branches are normal in caliber. No evidence of pleural effusion. Cardiac size appears normal. Visualized bones and soft tissues are unremarkable.

CONCLUSION: -

- **CT features are of complete collapse of right lung with extensive bronchiectasis, volume loss, mediastinal shift and hyper inflation of left lung with herniation across in right hemithorax favors Opportunistic infection in right lung. Clinical and lab correlation.**

Prepared By

Reported By

[redacted]
MBBS, FCPS
SR Paediatric Radiology

CamScanner
